# Enhanced Hsp70 Expression Protects against Acute Lung Injury by Modulating Apoptotic Pathways

**DOI:** 10.1371/journal.pone.0026956

**Published:** 2011-11-23

**Authors:** Gabriella Aschkenasy, Zohar Bromberg, Nichelle Raj, Clifford S. Deutschman, Yoram G. Weiss

**Affiliations:** 1 Department of Anesthesiology and Critical Care Medicine and the Goldyne Savad Institute of Gene Therapy, Hadassah-Hebrew University School of Medicine, Jerusalem, Israel; 2 Department of Anesthesiology and Critical Care and the Stavropoulos Sepsis Research Program, University of Pennsylvania School of Medicine, Philadelphia, Pennsylvania, United States of America; University of Illinois at Chicago, United States of America

## Abstract

The Acute respiratory distress syndrome (ARDS) is a highly lethal inflammatory lung disorder. Apoptosis plays a key role in its pathogenesis. We showed that an adenovirus expressing the 70 kDa heat shock protein Hsp70 (AdHSP) protected against sepsis-induced lung injury. In this study we tested the hypothesis that AdHSP attenuates apoptosis in sepsis-induced lung injury.

Sepsis was induced in rats via cecal ligation and double puncture (2CLP). At the time of 2CLP PBS, AdHSP or AdGFP (an adenoviral vector expressing green fluorescent protein) were injected into the tracheas of septic rats. 48 hours later, lungs were isolated. One lung was fixed for TUNEL staining and immunohistochemistry. The other was homogenized to isolate cytosolic and nuclear protein. Immunoblotting, gel filtration and co-immunoprecipitation were performed in these extracts. In separate experiments MLE-12 cells were incubated with medium, AdHSP or AdGFP. Cells were stimulated with TNFα. Cytosolic and nuclear proteins were isolated. These were subjected to immunoblotting, co- immunoprecipitation and a caspase-3 activity assay.

TUNEL assay demonstrated that AdHSP reduced alveolar cell apoptosis. This was confirmed by immunohistochemical detection of caspase 3 abundance. In lung isolated from septic animals, immunoblotting, co-immunoprecipitation and gel filtration studies revealed an increase in cytoplasmic complexes containing caspases 3, 8 and 9. AdHSP disrupted these complexes.

We propose that Hsp70 impairs apoptotic cellular pathways via interactions with caspases. Disruption of large complexes resulted in stabilization of lower molecular weight complexes, thereby, reducing nuclear caspase-3. Prevention of apoptosis in lung injury may preserve alveolar cells and aid in recovery.

## Introduction

The Acute Respiratory Distress Syndrome (ARDS) is a lethal, incompletely understood syndrome that frequently accompanies sepsis [1], [2]. The initial phase of the disorder involves unchecked inflammation that damages and may destroy type I alveolar epithelial cells [2], [3], [4]. While the exact mechanisms that lead to pulmonary cell death are unknown, it is likely that apoptosis plays an important pathogenic role [5], [6].

Normally, apoptosis is a homeostatic response to eliminate damaged or senescent cells [7], [8], [9]. This form of regulated cell death may be induced by a range of environmental, physical or chemical stresses [10]. Activation of apoptosis depends on a proteolytic system that involves some members of a family of intracellular enzymes called caspases. Several, such as caspase-3, -8 and -9, initiate and execute the cell death process while others have been implicated in inflammation [11], [12], [13]. Caspases are present in the cell in a precursor state. Following a pro-apoptotic signal, these pro-caspases are cleaved to yield the activated enzymes.

Activation and progression of apoptosis is tightly controlled. Part of the regulatory system involves Heat Shock Proteins (HSPs). These molecular chaperones are expressed both constitutively and in response to cellular and extracellular perturbations [14], [15]. As a result, they are involved in a myriad of normal homeostatic processes and can mediate cellular protection and recovery [16]. The involvement of HSPs in such a wide range of cellular activities reflects the ability of these molecules to interact with hydrophobic regions of nearly all proteins or polypeptides. Among HSPs, members of the 70 kDa subfamily, collectively referred to as Hsp70, are phylogenetically conserved, highly inducible and believed to play an essential role in normal cell processes and in the response to noxious stimuli [17].

Previously, we have demonstrated that intra-tracheal administration of an adenoviral vector that expresses Hsp70 (AdHSP) attenuates lung pathology and improves outcome in lung injury induced by cecal ligation and double puncture (2CLP) in rats [18], [19], [20]. In this model, AdHSP limited histologic lung injury, attenuated acute inflammation and neutrophil recruitment by suppression of NF-κB activation [21], limited over-proliferation of type II pneumocytes [22] and preserved type I alveolar epithelial cells [20]. This last finding may be related to altered apoptosis. Indeed, others have established that Hsp70 can attenuate apoptotic cellular pathways by preventing the recruitment of procaspase-9 to the Apaf-1 apoptosome, a complex composed of cytochrome C, oligomerized Apaf-1 and pro-caspase 9 [23]. Therefore, the studies outlined in this paper tested the hypothesis that AdHSP-induced expression of Hsp70 protects the lung from sepsis-induced injury in part by attenuating pro-apoptotic processes.

## Results

### AdHSP reduces apoptosis in 2CLP induced lung injury

**Figure 1:**
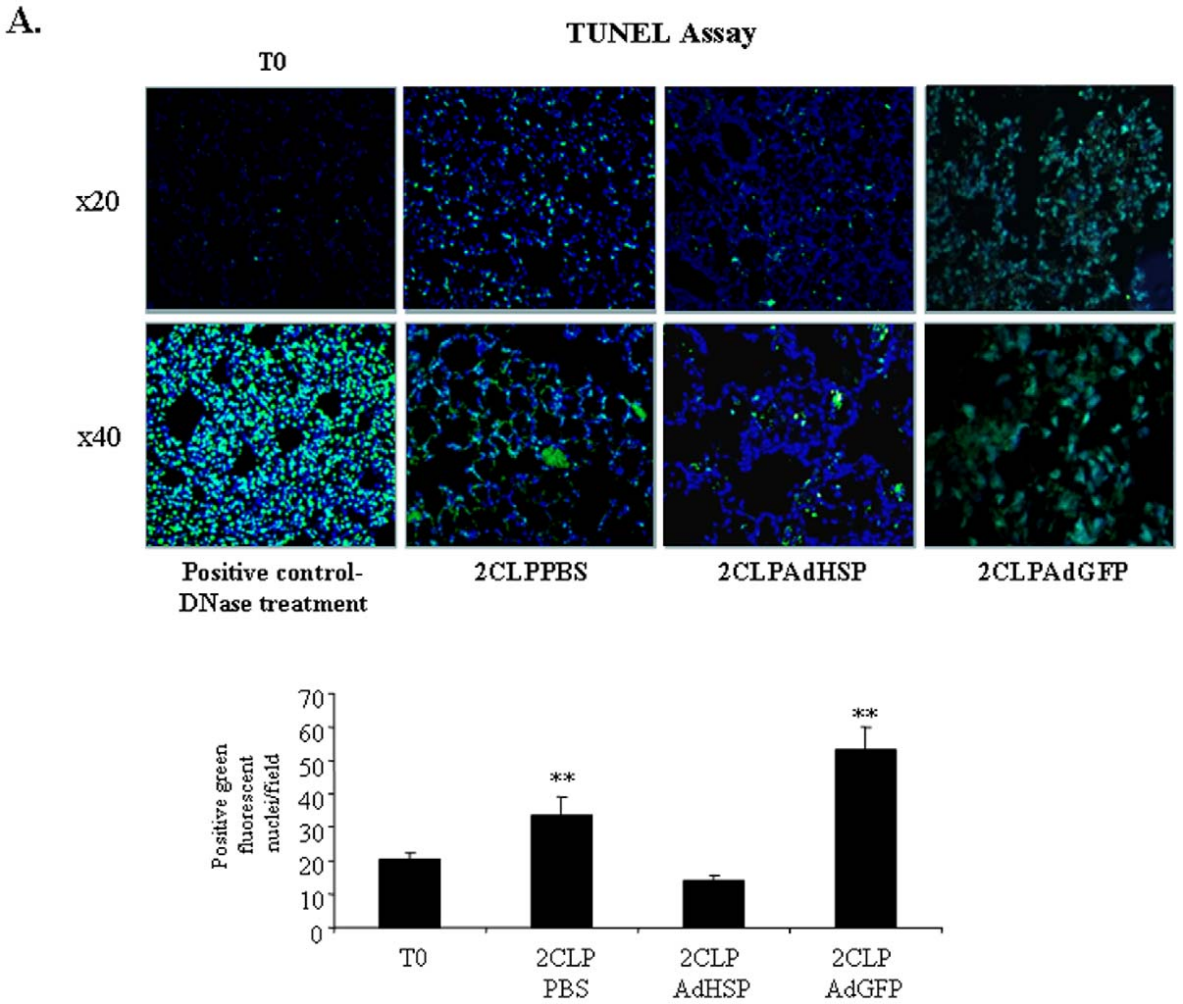
AdHSP reduces apoptosis in 2CLP induced ARDS. Representative TUNEL-stained fixed tissue section. Positive staining depicted in green. T0 - Untreated controls, 2CLPPBS - septic animals treated with PBS, 2CLPAdHSP - septic animals treated with AdHSP, 2CLPAdGFP – septic animals treated with AdGFP. Nuclei stained with DAPI. **Upper panel**: 20× magnification, **lower panel**: 40× magnification. Positive control - DNAse treatment. **Graph –** Graphic representation of fluorescent nuclei/low powered field. Quantification performed on 20 lung fields/slide at 20× magnification, five slides per animal and at least 3 animals for each intervention. Quantification of TUNEL staining was performed using Nikon software. 20 fields per slide were randomly manually selected by a single observer who was blinded to the intervention (AdHSP, AdGFP, PBS). Data are mean +/− standard deviation * = significantly different from T0 and AdHSP.

Previous publications investigating ARDS have demonstrated an association between inflammation and apoptosis [24]. In addition, we have shown that 2CLP-induced lung injury is associated with a loss of type I pulmonary epithelial (ATI) cells [20], [22]. Therefore, we used the TUNEL assay to test the hypothesis that treatment with AdHSP attenuated 2CLP-induced lung injury and apoptosis in these cells. Relative to normal, non-septic controls (T0), TUNEL staining (green fluorescence) was increased in PBS-treated (2CLP-PBS) (*P*<0.01) as well as in AdGFP-treated (2CLPAdGFP) rats. In contrast, lungs from septic rats treated with AdHSP had significantly less TUNEL staining than PBS (Fig. 1).

**Figure 2:**
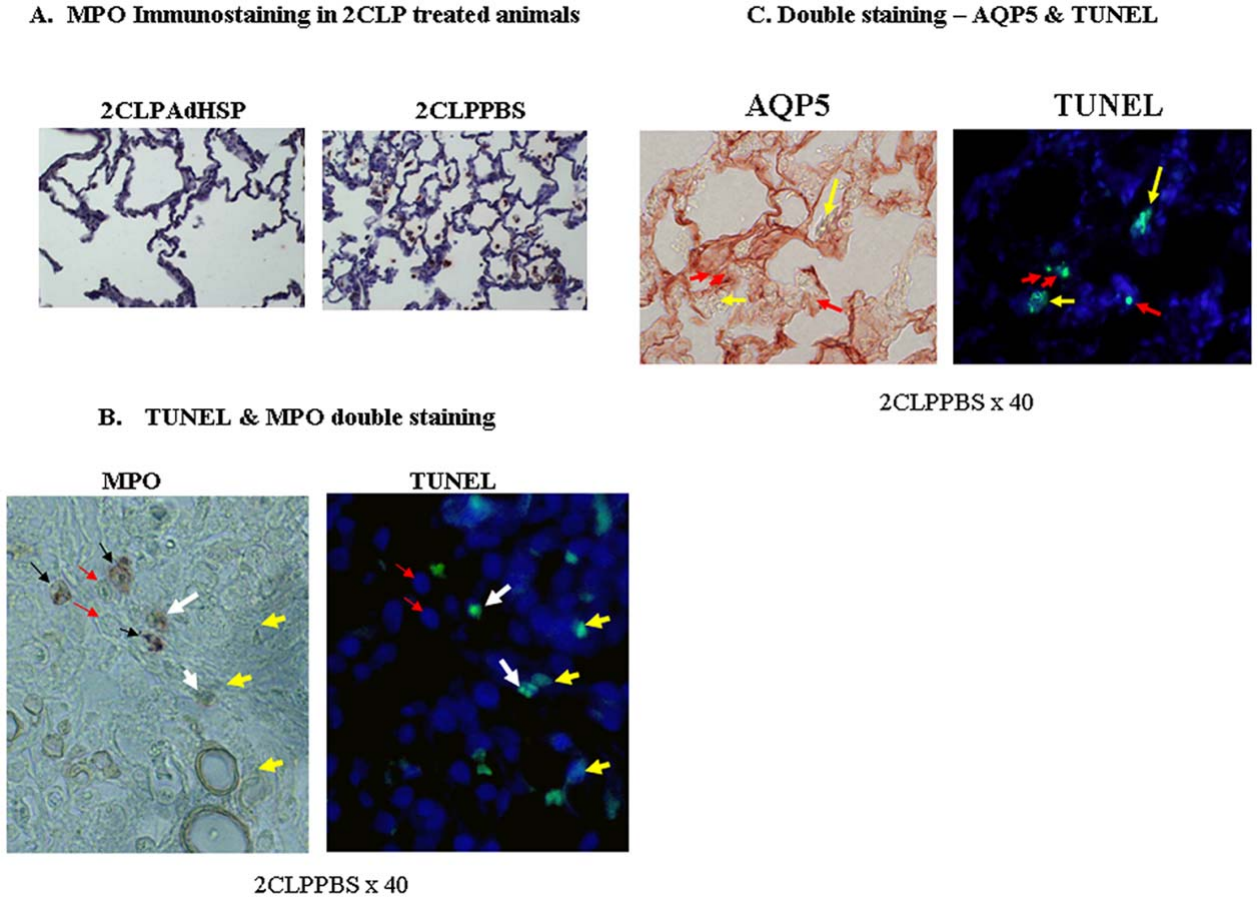
AdHSP reduces Myeloperoxidase (MPO) in 2CLP-induced lung injury. A. Myeloperoxidase (MPO) immunostaining. 20× Magnification. MPO containing cells stain brown. **Left panel**: 2CLPAdHSP, **Right panel**: 2CLPPBS. TUNEL and MPO double staining. 40× Magnification. **Left panel**: Myeloperoxidase (MPO) Immunostaining, MPO containing cells stain brown. **Right panel**: TUNEL fluorescent staining. TUNEL positive cells are green. White arrows depict apoptotic neutrophils – brown staining for MPO and green for TUNEL. Yellow arrows depict apoptotic cells that are negative for MPO but positive for TUNEL. Red arrows depict nuclei stained for dapi –showing the orientation of the slide. Black arrows depict MPO-positive cells that were not apoptotic. TUNEL and Aquaporin 5 (AQP5) double staining. AQP5 – a specific marker for alveolar type I cells. 40× magnification. Left panel: AQP5 immunostaining, AQP5 containing cell stain brown. Right panel: TUNEL fluorescent staining. TUNEL positive cells are green. Red arrows indicate positive staining of alveolar type I cells (both AQP5 and TUNEL). Yellow arrows indicate alveolar type II cells that are negatively stained for AQP5 and positively stained for TUNEL.

Under normal conditions the great majority of cells in the lung are type I pulmonary epithelial cells or endothelial cells. In ARDS, the normal cellular composition is altered by the infiltration of a large number of neutrophils. Thus, the enhanced TUNEL staining might reflect apoptosis in these inflammatory cells. To investigate this possibility and to strengthen our demonstration of an inflammatory process in 2CLP treated animals, Myeloperoxidase (MPO) immunostaining was performed. Data depict the presence of an increased number of neutrophils in the alveolar spaces of PBS-treated, as opposed to AdHSP-treated animals (Fig. 2A). Co-staining (Fig. 2B) for MPO (brown stain on left panel) and TUNEL (green stain on right panel) demonstrated that some of the TUNEL-positive cells were neutrophils (MPO positive, TUNEL positive, white arrows). However, most neutrophils (MPO positive, TUNEL negative, black arrows) were not apoptotic. In contrast, a number of cells (Fig. 2B, MPO negative, TUNEL positive, yellow arrows) were TUNEL positive but MPO negative. This indicates that the influx of neutrophils did not account for the enhanced apoptosis seen in untreated experimental lung injury. Double immunostaining to aquaporin-5 (AQP5), a key alveolar type I surface marker (20,22) and TUNEL, demonstrated that most of the apoptotosis (TUNEL positive, green fluorescence) occurred in alveolar type I (AQP5 positive, brown staining, red arrows) and type II (yellow arrows) (Fig. 2C). Thus, 2CLP-induced pulmonary apoptosis predominantly occurs in type I and type II alveolar epithelial cells and not in neutrophils.

### AdHSP reduces the abundance of Caspase 9 and Caspase 8 in 2CLP-induced lung injury

**Figure 3:**
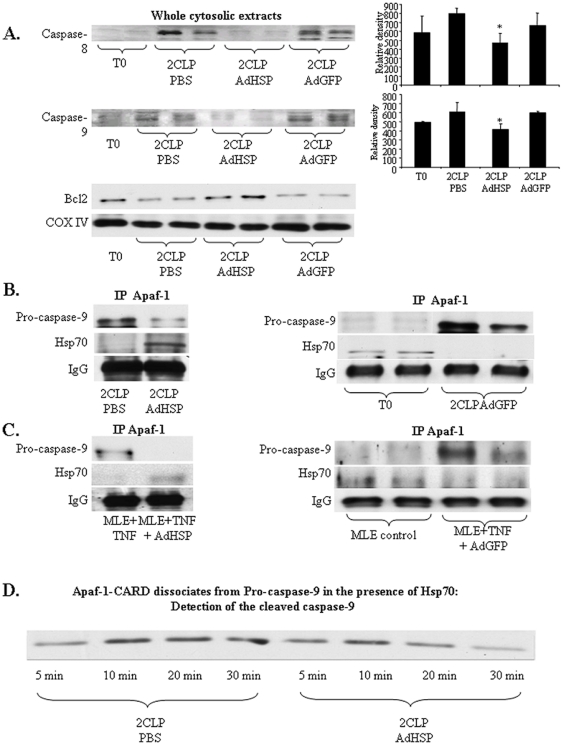
AdHSP reduces the abundance of caspases 8 and 9 in 2CLP-induced lung injury. A. Immunoblotting of whole cytosolic extracts for Caspase 8 and 9. Abbreviations as in Fig. 1. **Upper panel**: Representative autoradiogram of SDS-PAGE, 30 µg of cytosolic extract/lane. Primary rabbit polyclonal antibody to caspase 8. Secondary goat anti rabbit IgG. .Graphic representation of relative density of cytosolic caspase 8. Middle **panel**: Representative autoradiogram of SDS-PAGE, 30 µg of cytosolic extracts. Primary rabbit polyclonal antibody to caspase 9, secondary goat anti rabbit IgG. **Graph** - Graphic representation of relative density (mean +/− standard deviation) of cytosolic caspase 9. * = significantly different from 2CLPPBS and 2CLPAdGFP. Lower panels: Representative autoradiogram of SDS-PAGE, 30 µg of mitochondrial extract/lane. Primary mouse monoclonal antibody to Bcl2, secondary goat anti mouse IgG and primary mouse monoclonal antibody to COX IV, secondary goat anti mouse IgG. COX IV serves as mitochondrial loading control. **B. Hsp70** ***in vivo*** **interaction with apopotosomal Apaf-1.** Representative autoradiograms. Samples were immunoprecipitated with a rabbit polyclonal antibody to Apaf-1 and subjected to SDS-PAGE. Upper panels: Immunoblotting with a primary rabbit polyclonal antibody to pro-caspase 9, secondary goat anti rabbit IgG. Middle panels: Immunoblotting with primary mouse monoclonal antibody to Hsp70, secondary goat anti mouse IgG. Lower panels: IgG detection IgG serves as loading control. 250 µg of cytosolic extracts obtained from TO, 2CLPPBS, 2CLPAdHSP or 2CLPAdGFP treated animals sacrificed 48 hrs after the induction of sepsis. **C. Hsp70 in vitro, MLE-12 cells, interaction with apopotosomal Apaf-1.** Representative autoradiograms. 250 µg of cytosolic extracts obtained from non treated MLE-12 cells (controls), stimulated with tumor necrosis factor (TNF) and treated with AdHSP or AdGFP. Samples were immunoprecipitated with a rabbit polyclonal antibody to Apaf-1 and subjected to SDS-PAGE. Upper panels: Immunoblotting with a primary rabbit polyclonal antibody to pro-caspase 9, secondary goat anti rabbit IgG. Middle panels: Immunoblotting with primary mouse monoclonal antibody to Hsp70, secondary goat anti mouse IgG. Lower panels: IgG serves as loading control. **D. Apaf-1 – CARD dissociates from Pro-caspase-9 in the presence of Hsp70.** Representative autoradiograms. 100 µg of cytosolic extracts obtained from 2CLPPBS and 2CLAdHSP treated animals, were immunoprecipitated with Pro-caspase-9 and further incubated with GST-Apaf-1 – CARD obtained from BL-21 cells, together with 5 mM ATP and 5 µg/ml human Cytochrome C for 5, 10, 20 and 30 minutes. Samples were subjected to SDS-PAGE, immunoblotted and the membranes were incubated with primary rabbit polyclonal antibody to Cleaved Caspase-9, secondary to goat anti rabbit IgG.

Others have shown that Hsp70 affects the apoptosome [25]. However, the effects of Hsp70 on other pro-apoptotic enzymatic complexes have not been fully explored. Studies indicate that apoptosis initiated by an extracellular signal requires activation of caspase 8 while the mitochondrial pathway proceeds via caspase 9 [13]. Data examining the effects of AdHSP on the apoptotic cellular pathway during 2CLP-induced lung injury are presented in Fig. 3. Immunoblotting revealed that, relative to untreated controls (T0), treatment of with PBS or AdGFP increased the abundance of activated caspase 8 (upper panel) and caspase 9 (middle panel) in rat lung cytosolic extracts isolated 48 hrs after 2CLP (Fig. 3A). However, in extracts isolated 48 hours after 2CLP from rats treated with AdHSP, the cytosolic abundance of activated caspases 8 and 9 in the lungs was significantly lower than that observed in isolates obtained from similar animals treated with PBS or AdGFP (*P*<0.049, *P*<0.041 respectively).

Protein of the anti-apoptotic Bcl2 family play a key role in the regulation of mitochondria/apoptosome-dependent apoptosis [26]. To determine the effects of AdHSP on 2CLP-induced changes in Bcl2, we examined the mitochondrial Bcl2 abundance (Fig. 3A, lower panel). These data reveal preserved levels of Bcl2 in T0 and 2CLPAdHSP animals. However, treatment of 2CLP animals with PBS or AdGFP significantly reduced Bcl2 abundance. These data suggest that AdHSP exerts its effect, in part, by preserving Bcl2.

### Hsp70 disrupts the normal apoptotic signaling Apaf-1

An important study by Saleh et al [25] demonstrated that the anti-apoptotic effect of Hsp70 was mediated via a direct association with the caspase-recruitment domain of Apaf-1. This prevented the oligomerization of Apaf-1, the subsequent association of Apaf-1 with pro-caspase 9 and ultimately apoptosome formation. Other *in-vitro* studies revealed that Hsp70 directly prevented the recruitment of pro-caspase-9 to the Apaf-1 apoptosome [23]. These effects on caspase recruitment and apoptosome formation might be involved in Hsp70-mediated effects on sepsis-induced lung injury. Therefore, we examined the effects of AdHSP on the interaction between Apaf-1 and pro-caspase-9 in lung following 2CLP. Lung extracts were immunoprecipitated with an antibody directed at Apaf-1 and subjected to immunoblotting with an antibody to pro-caspase 9. Data are displayed in Fig. 3B. No interaction was noted in non-septic, untreated animals (T0). In septic animals treated with PBS, immunoblotting revealed a strong association between Apaf-1 and pro-caspase-9 (2CLPPBS). Similar results were observed in animals treated with AdGFP (2CLPAdGFP) However, this interaction was markedly reduced in animals treated with AdHSP (2CLPAdHSP). To strengthen our contention that these findings were likely specific to alveolar epithelial cells and not other cells types present in lung extracts, we performed *in-vitro* experiments where we stimulated MLE-12 cells (Type II pneumocyte analogs) with tumor necrosis factor (TNF). These cell cultured extracts were immunoprecipitated with anti-Apaf-1 and immunoblotting with an antibody to pro-caspase-9 was performed. Data are depicted in Fig. 3C. In cells treated with TNF alone (MLE+TNF), or with AdGFP and TNF stimulation (MLE+TNF+AdGFP) there is an association between Apaf-1 and pro-caspase 9. No Hsp70 was noted. When cells were incubated with AdHSP and then subjected to TNF stimulation (MLE+AdHSP+TNF) we found an association between Apaf-1 and Hsp70 but a less robust association of Apaf-1 with pro-caspase 9. However, similar experiments involving immunoblotting with an antibody to caspase-8 failed to demonstrate an interaction between Apaf-1 and caspase 8 (data not shown).

The apoptosome is a large (approximately 700–1400 kDa), caspase-activating complex that is assembled from Apaf-1 and caspase-9 when cytochrome c is released during mitochondrial-dependent apoptotic cell death. Apaf-1, the core scaffold protein, is approximately 135 kDa and contains a caspase recruitment domain (CARD) [27]. To examine the effects of AdHSP, we incubated GST-Apaf-1-CARD obtained from BL-21 cells, Cytochrome C and ATP, with immunopercipitated pro-caspase 9 isolated from 2CLPPBS and 2CLPAdHSP treated Lungs. Data depicted in Fig. 3D reveal increased cleaved casapase 9 production after the first five minutes of incubation in 2CLPPBS animals. In contrast, 2CLPAdHSP animals depict a decrease in cleaved caspase 9 production.

### AdHSP attenuates the expression of inactive and active caspase-3 in 2CLP-induced lung injury

**Figure 4:**
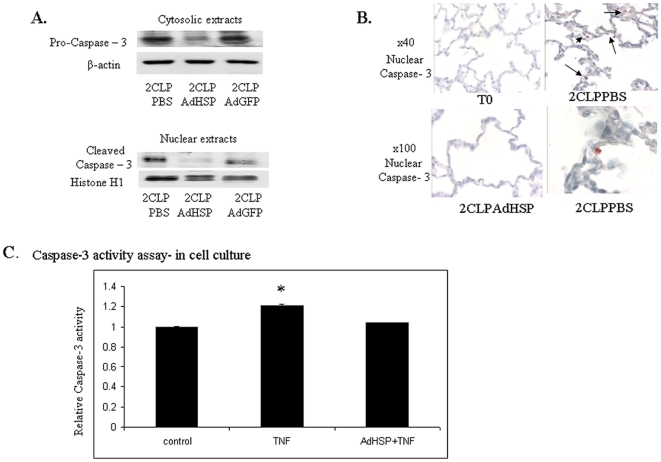
AdHSP prevents nuclear translocation of activated Caspase-3. A. Representative autoradiograms for pro-caspase 3 and activated (cleaved) Caspase 3. 30 µg of cytosolic (upper panels) and nuclear (lower panel) extracts were subjected to SDS-PAGE. Immunoblotting performed with primary rabbit polyclonal antibody to pro- caspase 3 and secondary goat anti rabbit IgG, primary goat antibody to β-actin and secondary donkey anti goat IgG, primary rabbit polyclonal antibody to active (cleaved) caspase-3 and secondary goat anti rabbit IgG, primary mouse monoclonal antibody to histone (H1) and secondary goat anti mouse IgG.. β-actin and histone serve as loading controls. **B. Representative stained fixed tissue section depicting intra-nuclear staining for Caspase 3.** Sections obtained from T0 control, 2CLPPBS and 2CLPHSP rats. Tissue isolated 48 hrs after the induction of sepsis. **Upper panel**: 40× magnifications. Black arrows indicate active caspase 3 stained nuclei. **Lower panel**: 100× magnification of upper panel. **C. Caspase 3 Activity Assay.** Graphic representation of relative caspase-3 enzymatic activity (mean +/− standard deviation) * = significantly different from Control and AdHSP+TNF.

Activation of caspase 3 is the terminal step in the apoptotic cascade and the main executioner of apoptosis [28]. The process involves cleavage of pro-caspase 3. This in turn is facilitated by interaction of the uncleaved, inactive pro-caspase-3 with either caspase-8 or caspase 9 that has been activated by the apoptosome. Association permits cleavage of pro-caspase-3 and nuclear transmigration of the now activated caspase-3. We sought to determine if 2CLP activated pro-caspase 3 cleavage and to examine the effects of Hsp70 on this process. Therefore we investigated the effects of AdHSP on 2CLP-induced changes in the cytosolic abundance of uncleaved (inactive) pro-caspase-3 and the intranuclear abundance of cleaved (active) caspase-3. Lung homogenate fractions were obtained from rats subjected to 2CLP and treated with either PBS (2CLPPBS), AdGFP (2CLPAdGFP) or AdHSP (2CLPAdHSP). Data are depicted in Fig. 4. 2CLP increased the abundance of pro-caspase-3 in the cytosol (Fig. 4a, upper panel) and cleaved caspase-3 in the nucleus (Fig. 4a, lower panel) of animals treated with PBS or AdGFP. In contrast, neither was increased in animals subjected to 2CLP and treated with AdHSP (Fig. 4a). To confirm these findings we immunostained lung sections with an antibody to cleaved caspase-3 (Fig. 4B). Relative to T0, caspase 3 staining increased in the nuclei of cells in sections obtained from septic animals treated with PBS (2CLPPBS). There was little staining, however, in rats treated with AdHSP at the time of 2CLP (2CLPAdHSP). The ×100 magnification depicts the nuclear staining (Fig. 4B Lower panel). The amount of staining is depicted at lower magnification (Fig. 4B Upper panel). These findings further substantiate the ability of Hsp70 to attenuate caspase-3 cleavage and nuclear translocation and thus activation. To further solidify our findings we measured Caspase-3 activity directly in TNF – activated MLE-12 cells (Fig. 4c). These data indicate that cytoplasmic caspase-3 activity was decreased by AdHSP treatment. Unfortunately, it was not possible to measure caspase-3 activity in lung extracts *in – vivo* because of the lack of a specific assay kit for homogenates/tissues.

### Multiple effects of AdHSP on 2CLP-induced caspase complexes - disruption and stabilization

**Figure 5:**
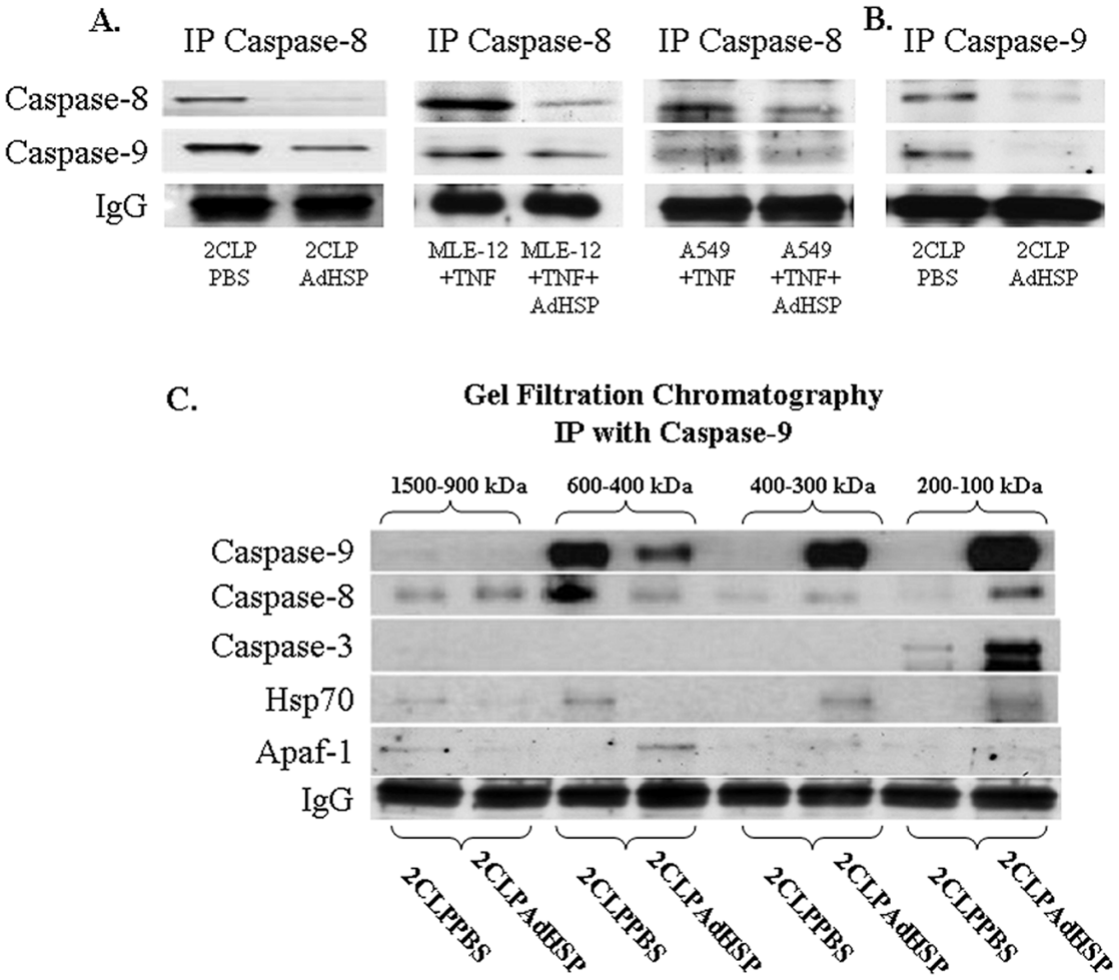
AdHSP alters interactions between Caspase 8, 9, 3 and Apaf-1. A and B: Representative Autoradiogram Demonstrating AdHSP treatment disrupts interaction between caspase 8 and caspase 9. Representative autoradiograms. 250 µg of cytosolic extracts were immunoprecipitated with rabbit polyclonal antibody to caspase 8 or caspase-9 and subjected to SDS-PAGE. Immunoblotting performed with either primary rabbit polyclonal antibodies to caspase 9 or caspase-8, secondary goat anti rabbit IgG. Lower panels: IgG detection. IgG serves as loading control. Abbreviations as in Figure 1. **C: Hsp70 disrupts caspases 3, 8 9 and Apaf-1 complexes.** 250 µg of cytosolic extracts from lung tissue fractionated via column chromatography, eluted by molecular weight, immunoprecipitated with an antibody to caspase-9 and subjected to 9% SDS-PAGE. Molecular weight of each fraction (kDa) indicated at the top of the figure. Detecting antibodies (anti-caspase 9, anti-caspase 8, anti-pro-caspase 3, anti-Hsp70 and anti-Apaf-1,) noted to the left of the panels. Lower panel: IgG detection. IgG serves as loading control. Abbreviations as in Figure 1.

In a previous paper we have shown that over-expression of Hsp70 disrupted large molecular weight complexes [21]. The same effect may limit the formation of large active molecular weight complexes associated with the apoptotic pathway. Caspase 3 is activated by either caspase 8 (extrinsic pathway) or caspase 9 (intrinsic/mitochondrial pathway). In either pathway, *in vitro* studies have demonstrated that caspase 3 forms a complex with the activating protease [29]. However, this finding has not been confirmed in complex *in vivo* situations such as sepsis-induced lung injury. Therefore we used co-immunoprecipitation and immunoblotting to examine the effects of AdHSP on the sepsis-induced formation of complexes containing pro-caspase 3, caspase 8 and caspase 9, as well as Apaf-1. Cytosolic lung extracts obtained 48 hrs after 2CLP were immunoprecipitated with an antibody to caspase-9 and subjected to immunoblotting with antibodies to caspases-8, 9, 3 and Apaf-1 (Fig. 5). These data revealed the previously reported interactions between caspases-8 and -3 and caspases-9 and -3 as well as an unexpected and previously unreported cytosolic interaction between caspases-8 and -9 (Fig. 5A,B,C). Immunoprecipitation of samples with an antibody to caspse-8 followed by immunoblotting with an antibody to caspase-9 and, conversely, immunoprecipitation with and antibody to caspase 9 followed by immunoblotting with anti- caspase 8 confirmed this finding (Fig. 5A,B).

Treatment with AdHSP significantly altered this association between caspase 8 and caspase 9. Similar effects were found in TNF-activated MLE-12 and A549 cells (Fig. 5A). Further, an association between caspase 8 and caspase 9 was detected in 2CLP lungs *in-vivo*. (Fig. 5B).

To further investigate the effect of AdHSP on the association between these enzymes, we immunoprecipitated cell homogenates with an antibody to caspase 9 and performed fractionation by molecular weight (gel filtration) followed by immunoblotting. These studies (Fig. 5C) revealed that the interaction between caspases 8 and 9 found in PBS-treated septic rats (2CLPPBS) occurred in complexes with molecular weights between 400–600 kDa that contained little caspase 3 or Hsp-70. Treatment with AdHSP disrupted the caspase 8–9 interaction and shifted caspase-9 abundance to smaller (100–400 kDa) complexes that contained Hsp70 and Caspase-3 as well as a more limited amount of caspase 8. Finally, an Apaf-1/caspase-9 complex, shifted from the 1500–900 kDa fraction in 2CLPPBS to the 600–400 kDa in the 2CLPADHSP fraction. These data are consistent with an Hsp70-mediated stabilization of low molecular weight cytosolic complexes containing primarily caspase 9 as well as varying amounts of caspase 3 and Hsp70. This is similar to and effect on NF-κB complexes that we have reported previously [21], [22].

## Discussion

Our previous studies have demonstrated that AdHSP-induced augmentation of Hsp70 abundance protects against sepsis-induced acute lung injury [19]. Our subsequent work has sought to identify the mechanisms by which the attenuation of pulmonary damage might occur and to examine the therapeutic role of Hsp70. These data have revealed that AdHSP interferes with the activation of the pro-inflammatory transcription factor NF-κB [21] and limits division of type II pulmonary epithelial cells [22]. Enhanced apoptosis also has been implicated in the development of acute lung injury. Others have demonstrated that Hsp70 attenuates apoptosis *in-vitro* [25], [30], [31], [32] but this has never been explored in an *in vivo* model of an acute disease state. Therefore, it is logical to examine the effects of AdHSP on apoptotic cellular pathways in our lung injury model.

As expected, lung injury induced by 2CLP increased the abundance of active caspase 8, active and inactive caspase 9 and pro-caspase 3. However, treatment with AdHSP attenuated this activation and decreased the abundance of these enzymes to levels equal or close to those observed in unoperated, non-septic controls. This effect is not the result of viral infection, as treatment with AdGFP, an adenoviral vector expressing the marker green fluorescent protein, did not affect sepsis-induced apoptosis in pulmonary cells.

Our previous work has demonstrated that Hsp70 alters inflammation and cell replication in the lungs of septic rats by associating with key enzymes and effecting protein-protein interactions within hetero-multimeric complexes [21], [22]. Others have demonstrated that Hsp70 has the capacity to protect certain caspase substrates by directly binding to caspase-3 and caspase-9 and thereby, promote survival [33].

The experiments described here reveal a similar mechanism. Specifically, AdHSP alters interactions between caspases 8, 9 and 3. As in previous studies, the experiments detailed here confirm the multi-faceted nature of these Hsp70-mediated effects. *In vivo*, Hsp-70 disrupted the high molecular weight caspase complex and stabilized caspase 3 in a low molecular complex with caspase 8 and 9. In TNF-treated MLE-12 cells, treatment with AdHSP blocked activation of caspase-3. AdHSP also limited nuclear translocation of caspase-3 by stabilizing the caspase 8/9-caspase 3 complex and preventing caspase-3 activation. We present reduced pro-capase 3 levels in 2CLPAdHSP treated animals. Most papers address specifically the cleaved caspase 3 without evaluating possible effects of inflammatory processes on pro-caspase 3 levels. As shown here (Fig. 4A), also others have shown that pro-caspase 3 levels increase following inflammatory processes such as renal injury [34] and decrease after caspase-3 siRNA addition within the injured kidney [35]. We have observed similar differential effects in Hsp70's interaction with elements of the NF-κB signaling pathway [21]. Others investigating apoptosis in septic lung injury in the 2CLP model found that heat shock preconditioning similarly inhibits polymorphonuclear leukocyte apoptosis by decreasing caspase-3 activity [36]. *In vitro* studies also demonstrate that Hsp70 prevented recruitment of pro-caspase-9 to the Apaf-1 apoptosome [23], [25], [37]. Furthermore, using GST-Apaf-1-CARD we demonstrate that Apaf-1-CARD dissociates from pro-caspase 9 in the presence of HSP-70 resulting in reduced production of caspase 9. Our data confirmed these findings *in vivo and ex-vivo*. Finally, the experiments presented here uniquely demonstrate that Hsp70 impairs protein interactions, in pulmonary epithelial cells, that effect apoptotic cellular pathways through mechanisms not directly involving the apoptosome, thus modulating apoptosis in several independent ways. The relative importance of each of the observed interactions in mediating the effects of Hsp70 on apoptosis will require further investigation as will the role of other, non-Hsp70 effects. Morimoto has pointed that Hsp70-protein interactions likely are modulated by associated molecules called co-chaperones [38]. This suggests that Hsp70-protein interactions may be different in other cell types and under alternate *in vivo* conditions.

It is possible that Hsp70 does not disrupt but rather prevents apoptosome formation. However, we have shown that adenoviral treatment with a marker protein or with HSP do not lead to significant increases in Hsp70 abundance until 16–24 hours after administration [19], [20]. Changes consistent with lung injury are noted as early as 6 hours after 2CLP [20]. Therefore, disruption is more likely.

One important observation from our previous studies involves stabilization of proteins into complexes that alter activity. Specifically, our investigations into the proteins involved in the activation of NF-κB have revealed Hsp70-mediated stabilization of low molecular weight complexes. These are known to have lower activity than larger complexes containing more enzymatically active subunits [21]. In the data presented here, we have uncovered interactions involving several combinations of caspase-9, 8 and 3 in septic animals treated with PBS. These complexes appear to have been disrupted by enhanced Hsp70 expression. Further, as in the NF-κB pathway, AdHSP appears to disrupt high molecular caspase complexes and to stabilize low-molecular intermediate complexes. As a result, it is likely that Hsp70 prevents Caspase-3 migration into the nucleus. This suggests one common mechanism by which Hsp70 alters enzymatic activity. Further investigations should be directed towards examination of this possibility.

In summary, the studies detailed here demonstrate Hsp70-mediated effects on apoptosis. In concert with our previous work on Hsp70 and the NF-κB inflammatory pathway [21] and on cell replication [22], they expand our understanding of the mechanisms by which Hsp70 alters cellular processes. Further, they pave the way for therapeutic use of Hsp70 in the treatment of ARDS, a common, life-threatening syndrome.

## Materials and Methods

### Induction of Sepsis

All studies were approved by the Institutional Animal Care and Use Committees (IACUC) of both collaborating institutions and conform to both University Laboratory Animal Resources (ULAR), The Hebrew University School of Medicine ethics committee – research number MD 109.14-4 and National Institute of Health standards. Severe sepsis was induced by cecal ligation and double puncture (2CLP) in male adolescent Sprague-Dawley rats under isoflurane anesthesia as previously described [18], [19], [20], [21], [22]. Animals were fluid resuscitated (50 cc/kg of 0.9% saline injected subcutaneously) at the time of surgery and at 24 hours. 48 hours after 2CLP animals were euthansized with an overdose (150 mg/kg) of pentobarbital. As previously described [18], [19], [20], [21], one lung was removed *en bloc*, perfused, gently inflated to a pressure of 20 cm H_2_O, fixed overnight in 10% formalin, sagitally sliced and paraffin embedded. Studies were performed on 5 µm sections. The other lung was homogenized for extraction of cytoplasmic and nuclear protein.

### Adenoviral Vector Administration

As previously described, 10^11^ viral plaque-forming units (PFUs) of either AdHSP or of a recombinant E1,E3-deleted adenoviral vector expressing Green Fluorescent Protein (AdGFP) dissolved in PBS (total volume, 300 µl) were administered via tracheal puncture at the time of 2CLP [18], [19]. Administration of a control vector, AdGFP, was used to provide data on the effects of an adenoviral vector that did not express HSP70. A second set of control animals were subjected to 2CLP and treated with intratracheal PBS alone.

### Isolation and Preparation of Cytosolic and Nuclear Extracts from Lungs

Cytosolic and nuclear proteins were isolated from lung tissue as previously described [18], [19]. Protein concentration was determined in an aliquot of each lysate using the Bradford method (Bio-Rad laboratories, GmbH, Manheim, Germany).

### Cell culture

MLE (murine lung epithelial)-12 cells and A549 lung carcinoma cells (ATCC, Manassas, VA, USA) were grown to confluence in Hites or RPMI mediums respectively, containing 10% FCS, 100 units/ml penicillin and 100 µg/ml streptomycin (Gibco BRL, Grand Island, NY, USA). After incubation with either PBS, AdGFP or AdHSP (both at 5×10^7^ PFU/ml) for 24 hrs, cells were treated with 20 ng/ml TNFα for 30 minutes (R&D, Minneapolis, USA). The cells were extracted as previously described [21]. Cytosolic extracts were subjected to immunoprecipitation using a rabbit polyclonal antibody to Apaf-1 (diluted 1∶200), rabbit polyclonal caspase-8 (diluted 1∶200) or to enzymatic activity assay as described below.

### Immunoblot Analysis

30 µg of total protein lysate were separated using 9% SDS-PAGE. All signals were detected by enhanced chemiluminescence and quantified by scanning densitometry. Caspases 8, 9, 3 and Apaf-1 (diluted 1∶500) were identified using primary polyclonal rabbit antibodies (Santa-Cruz Biotechnology Inc., Santa-Cruz, CA, USA). Hsp70 (diluted 1∶1000) was detected with either a mouse monoclonal or rabbit polyclonal antibody (StressGen Biotechnologies Corp, Canada). Bcl-2 (diluted 1∶1000) was detected with mouse monoclonal antibody (MBL Inc, Woburn, MA). β-actin (diluted 1∶15,000) was detected with goat antibody (Sigma, St. Louis, MO). Histone (H1) (diluted 1∶1000) was detected with a mouse monoclonal antibody (Santa-Cruz Biotechnology Inc., Santa-Cruz, CA, USA). COX IV (mitochondria loading control) (diluted 1∶1000) was detected with a mouse monoclonal antibody (abcam, Cambridge, UK). The secondary antibodies were goat anti-rabbit IgG, goat anti mouse IgG or donkey anti goat IgG (Jackson, Immunoresearch Lab., Inc., West Grove, PA., USA).

### Immunoprecipitation

250 µg of cytosolic extract were immunoprecipitated using a rabbit polyclonal anti-caspase 9, 8 or rabbit polyclonal anti-Apaf-1 (Santa Cruz Biotech Inc), diluted either 1∶500 or 1∶200. Samples were agitated overnight at 4°C. Protein A/G beads (Sigma, St. Louis, MO, USA and Pierce Inc., USA) were added and the samples were agitated for two more hours at 4°C and centrifuged at 14,000 rpm for 5 min at 4°C. The resulting pellet was washed three times with lysis buffer [21], [39], suspended in sample buffer containing 5 M urea or beta-mercaptoethanol and heated to 60°C or boiled for 5 min respectively. The resulting mixture was subjected to immunoblotting as described above.

### Immunohistochemistry of lung tissue

Immunostaining was performed as previously described [19], [20] using primary rabbit polyclonal antibody to myeloperoxidase (MPO), (Neomarker, Fremont, CA), diluted 1∶100, rabbit polyclonal antibody to Aquaporin 5 (AQP5) (Almone Inc., Jerusalem, Israel), diluted 1∶100 and a primary rabbit antibody directed at the cleaved form of caspase-3 (Cell Signaling Technology Inc. Beverly, MA, USA). MPO is a marker for neutrophils [40]. For detection we used an anti rabbit IgG (EnVision System, DAKO, Carpinteria, CA, USA) conjugated to horseradish peroxidase as previously described [21], [41].

### Gel Filtration Chromatography (Fractionation by molecular weight)

0. 5 ml of cytosolic extracts were loaded onto a Sephacryl S300 filtration column (Amersham Pharmacia- Biotech, Uppsala, Sweden). The gel filtration buffer contained: 20 mM Tris (pH 8.0), 0.1 M NaCl and 0.02% NaN_3_. Proteins were eluted from the column at a flow rate of 1 ml/min and 1 ml fractions were collected. The column was calibrated using the following standards: Thyroglobulin (669 kDa), ferritin (440 kDa), catalase (232 kDa), aldolase (163 kDa), bovine serum albumin (67 kDa), ovoalbumin (44 kDa) and myoglobulin (17 kDa). Isolated gel filtration chromatography fractions were loaded onto 8% or 9% gels, subjected to SDS-PAGE and transferred to nitrocellulose membranes.

### TUNEL Assay

Fragmented DNA in apoptotic cells was quantified using the Dead End™ Fluorometric TUNEL System (Promega Corp., Madison, WI, USA) on paraffin-embedded sections. Fluorescein-12-dUTP-labeled DNA was visualized directly using fluorescence microscopy. Nuclei were counterstained with DAPI (Vector Laboratories Inc, Burlingame, CA, USA) [42]. For apoptotic cells, we examined positive green fluorescent apoptotic nuclei in 20 low power fields/slide at 20× magnification 20 fields per slide were randomly manually selected by a single observer who was blinded to the intervention (AdHSP, AdGFP, PBS).

### Combined immunoperoxidase or Aquaporin staining and TUNEL

In order to characterize the apoptotic cells within the septic lung, a double labeling technique was used. We first stained with a Chromogen AEC (EnVision System, DAKO, Carpinteria, CA, USA) method to detect MPO in neutrophils or Aquaporin (AQP5) in alveolar type I cells and then with TUNEL to indentify apoptosis. MPO was detected using a primary rabbit polyclonal antibody (Neomarker, Fremont, CA) diluted 1∶100 and a secondary anti rabbit IgG. AQP5 was detected using a primary rabbit polyclonal antibody (Almone Inc., Jerusalem, Israel) diluted 1∶100 and a secondary anti rabbit IgG. After washing in phosphate buffered saline (PBS), the TUNEL assay was performed as previously described [43] as well as the method described above.

### Caspase -3 enzymatic activity assay

Caspase-3 enzymatic activity was quantified using a colorimetric activity assay (Chemicon international Inc, USA & Canada). Briefly, apoptosis was induced in MLE-12 cells using 20 ng/ml TNFα for 30 minutes. 1×10^6^ cells were subjected to centrifugation at 1500 rpm for 10 minutes. Cells were resuspended in cold lysis buffer, incubated for 10 minutes on ice and centrifuged for 5 minutes at 10,000 g. Supernatants (cytosolic extracts) were incubated with an assay mixture containing caspase-3 substrate and assay buffer (final concentration of 1×) for 1 hr at 37°C. Optical density at 405 nm was determined.

### *Ex-vivo* GST-Apaf-1 CARD competitive assay

GST-Apaf-1 CARD plasmid (1–97 aa) was transformed into BL-21 competent cells. Cells were grown, lysed and incubated with glutathione beads over night at 4°C followed by incubation with glutathione reduced buffer, as previously described [21]. The supernatant was further incubated with Co-immunoprecipitated lung extracts with Pro-caspase-9 antibody as previously described [21]. 5 mM ATP and 5 µg/ml of human Cytochrome-C recombinant protein were added to the mixture for 5, 10, 20 and 30 minutes at 37°C. Sample buffer was added to the samples, the samples were boiled for 5 minutes at 95°C, centrifuged and the supernatants were loaded on 8% SDS-PAGE. Nitrocellulose membranes were further incubated with Cleaved Caspase-9 antibody.

### Statistical analysis

At least three independent samples were evaluated for each experimental point. Analysis of variance (ANOVA) with Bonferroni's correction was used to examine differences between and within groups. The significance level was set at *P*<0.05.
